# *catena*-Poly[[triphenyl­tin(IV)]-μ-3-methyl­phenyl­seleninato-κ^2^ *O*:*O*′]

**DOI:** 10.1107/S1600536811049245

**Published:** 2011-11-23

**Authors:** Jing Ru, Rufen Zhang

**Affiliations:** a College of Chemistry and Chemical Engineering, Liaocheng University, Shandong 252059, People’s Republic of China

## Abstract

In the polymeric title coordination compound, [Sn(C_6_H_5_)_3_(C_7_H_7_O_2_Se)]_*n*_, the Sn^IV^ atom has a distorted trigonal–bipyramidal geometry, with two O atoms from two symmetry-related bridging seleninate ligands in axial positions and three phenyl groups in the equatorial plane. In the crystal, the complex exhibits a zigzag chain structure running parallel to the *c* axis. An intra­chain C—H⋯O hydrogen bond is observed.

## Related literature

For the biological activity of organotin compounds, see: Dubey & Roy (2003 ▶). For related structures, see: Chandrasekhar *et al.* (1992 ▶); Guo *et al.* (2011 ▶).
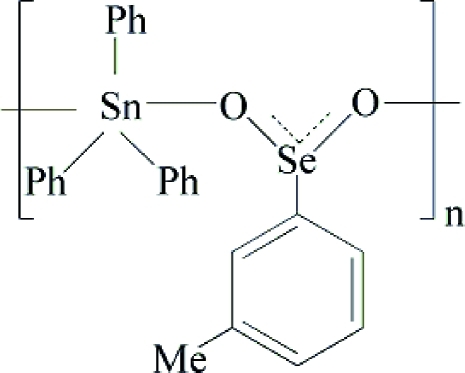


## Experimental

### 

#### Crystal data

[Sn(C_6_H_5_)_3_(C_7_H_7_O_2_Se)]
*M* *_r_* = 552.08
Monoclinic, 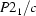

*a* = 12.3293 (11) Å
*b* = 14.3519 (16) Å
*c* = 12.2865 (13) Å
β = 94.324 (1)°
*V* = 2167.9 (4) Å^3^
*Z* = 4
Mo *K*α radiation
μ = 2.88 mm^−1^
*T* = 298 K
0.35 × 0.14 × 0.10 mm

#### Data collection

Bruker SMART 1000 CCD area-detector diffractometer
Absorption correction: multi-scan (*SADABS*; Bruker, 2007 ▶) *T*_min_ = 0.433, *T*_max_ = 0.762
10632 measured reflections
3821 independent reflections
2802 reflections with *I* > 2σ(*I*)
*R*_int_ = 0.061

#### Refinement

*R*[*F* ^2^ > 2σ(*F* ^2^)] = 0.038
*wR*(*F* ^2^) = 0.097
*S* = 1.09
3821 reflections
263 parameters
H-atom parameters constrained
Δρ_max_ = 1.11 e Å^−3^
Δρ_min_ = −0.71 e Å^−3^

### 

Data collection: *SMART* (Bruker, 2007 ▶); cell refinement: *SAINT* (Bruker, 2007 ▶); data reduction: *SAINT*; program(s) used to solve structure: *SHELXS97* (Sheldrick, 2008 ▶); program(s) used to refine structure: *SHELXL97* (Sheldrick, 2008 ▶); molecular graphics: *SHELXTL* (Sheldrick, 2008 ▶); software used to prepare material for publication: *SHELXTL*.

## Supplementary Material

Crystal structure: contains datablock(s) I, global. DOI: 10.1107/S1600536811049245/rz2663sup1.cif

Structure factors: contains datablock(s) I. DOI: 10.1107/S1600536811049245/rz2663Isup2.hkl

Additional supplementary materials:  crystallographic information; 3D view; checkCIF report

## Figures and Tables

**Table 1:** Hydrogen-bond geometry (Å, °)

*D*—H⋯*A*	*D*—H	H⋯*A*	*D*⋯*A*	*D*—H⋯*A*
C9—H9⋯O1	0.93	2.57	3.487 (7)	169

**Fig. 1.**
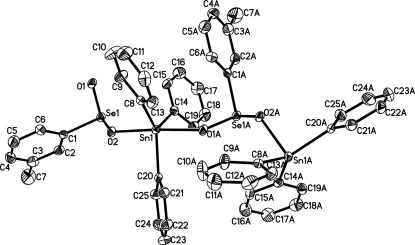
The asymmetric unit of the title compound, showing 50% probability displacement ellipsoids. H atoms have been omitted for clarity.

**Fig. 2.**
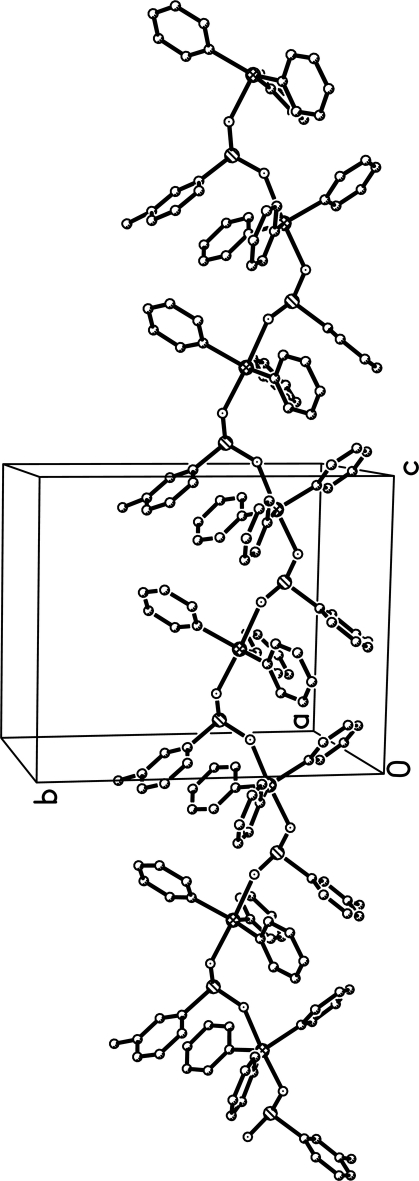
View of the one-dimensional zigzag chain structure running parallel to the c axis in the title compound. H atoms have been omitted for clarity.

## supplementary crystallographic information

### Comment

Organotin compounds have been attracting more and more attention due to their wide range of industrial applications and biological activities (Dubey & Roy, 2003). As a part of our ongoing investigations in this field (Guo *et al.*, 2011), we have synthesized the title compound and present its crystal structure herein.

The asymmetric unit of the title compound is shown in Fig. 1. An extended one-dimensional zigzag chain structure running parallel to the *c* axis is formed by the bridging role of the 3-methylphenylseleninate anions (Fig. 2). The Se—O bond distances in the compound (Se1—O1 = 1.674 (4) Å; Se1—O2 = 1.698 (3) Å) are comparable to those found in a related polymeric organotin complex (Chandrasekhar *et al.*, 1992). The Sn atom is five-coordinate in a slightly distorted trigonal-bipyramidal coordination geometry, provided by the phenyl groups in the equatorial positions and two O atoms of symmetry related 3-methylphenylseleninate groups in the axial positions. An intrachain C—H···O hydrogen bond is observed (Table 1).

### Experimental

The reaction was carried out under a nitrogen atmosphere. 3-Tolueneseleninic acid (1 mmol) and sodium ethoxide (1 mmol) were added to a stirred solution of methanol (30 ml) in a Schlenk flask and stirred for 30 min. Triphenyltin chloride (1 mmol) was then added to the reactor and the reaction mixture was stirred for 10 h at room temperature. The resulting clear solution was evaporated under vacuum. The product was crystallized from a solution of ether to yield colourless blocks of the title compound (yield 60%). Anal. Calcd (%) for C_25_H_22_O_2_Sn_1_Se_1_ (Mr = 552.08): C,54.39; H, 4.02. Found (%): C, 54.25; H, 4.28.

### Refinement

The H atoms were positioned geometrically, with methyl C—H distances of 0.96 Å and aromatic C—H distances of 0.93 Å, and refined as riding on their parent atoms, with *U*_iso_(H) = 1.5*U*_eq_(C) for the methyl group or *U*_iso_(H) = 1.2*U*_eq_(C) for the phenyl groups.

### Crystal data

**Table:**

[Sn(C_6_H_5_)_3_(C_7_H_7_O_2_Se)]	*F*(000) = 1088
*M**_r_* = 552.08	*D*_x_ = 1.691 Mg m^−^^3^
Monoclinic, *P*2_1_/*c*	Mo *K*α radiation, λ = 0.71073 Å
Hall symbol: -P 2ybc	Cell parameters from 3292 reflections
*a* = 12.3293 (11) Å	θ = 2.7–27.0°
*b* = 14.3519 (16) Å	µ = 2.88 mm^−^^1^
*c* = 12.2865 (13) Å	*T* = 298 K
β = 94.324 (1)°	Block, colourless
*V* = 2167.9 (4) Å^3^	0.35 × 0.14 × 0.10 mm
*Z* = 4	

### Data collection

**Table:**

Bruker SMART 1000 CCD area-detector diffractometer	3821 independent reflections
Radiation source: fine-focus sealed tube	2802 reflections with *I* > 2σ(*I*)
graphite	*R*_int_ = 0.061
phi and ω scans	θ_max_ = 25.0°, θ_min_ = 2.7°
Absorption correction: multi-scan (*SADABS*; Bruker, 2007)	*h* = −14→14
*T*_min_ = 0.433, *T*_max_ = 0.762	*k* = −17→17
10632 measured reflections	*l* = −14→12

### Refinement

**Table:**

Refinement on *F*^2^	Primary atom site location: structure-invariant direct methods
Least-squares matrix: full	Secondary atom site location: difference Fourier map
*R*[*F*^2^ > 2σ(*F*^2^)] = 0.038	Hydrogen site location: inferred from neighbouring sites
*wR*(*F*^2^) = 0.097	H-atom parameters constrained
*S* = 1.09	*w* = 1/[σ^2^(*F*_o_^2^) + (0.0361*P*)^2^ + 1.7781*P*] where *P* = (*F*_o_^2^ + 2*F*_c_^2^)/3
3821 reflections	(Δ/σ)_max_ = 0.001
263 parameters	Δρ_max_ = 1.11 e Å^−^^3^
0 restraints	Δρ_min_ = −0.71 e Å^−^^3^

### Special details

**Table:**

Geometry. All e.s.d.'s (except the e.s.d. in the dihedral angle between two l.s. planes) are estimated using the full covariance matrix. The cell e.s.d.'s are taken into account individually in the estimation of e.s.d.'s in distances, angles and torsion angles; correlations between e.s.d.'s in cell parameters are only used when they are defined by crystal symmetry. An approximate (isotropic) treatment of cell e.s.d.'s is used for estimating e.s.d.'s involving l.s. planes.
Refinement. Refinement of *F*^2^ against ALL reflections. The weighted *R*-factor *wR* and goodness of fit *S* are based on *F*^2^, conventional *R*-factors *R* are based on *F*, with *F* set to zero for negative *F*^2^. The threshold expression of *F*^2^ > σ(*F*^2^) is used only for calculating *R*-factors(gt) *etc*. and is not relevant to the choice of reflections for refinement. *R*-factors based on *F*^2^ are statistically about twice as large as those based on *F*, and *R*- factors based on ALL data will be even larger.

### Fractional atomic coordinates and isotropic or equivalent isotropic displacement parameters (Å^2^)

**Table:**

	*x*	*y*	*z*	*U*_iso_*/*U*_eq_	
Sn1	0.70340 (3)	0.19810 (3)	0.84922 (3)	0.02761 (13)	
Se1	0.77442 (5)	0.15274 (4)	0.58249 (4)	0.03037 (17)	
O1	0.7026 (3)	0.2382 (3)	0.5185 (3)	0.0369 (10)	
O2	0.7000 (3)	0.1284 (3)	0.6898 (3)	0.0358 (10)	
C1	0.7357 (5)	0.0477 (4)	0.4897 (4)	0.0301 (13)	
C2	0.8174 (5)	−0.0095 (4)	0.4585 (5)	0.0391 (15)	
H2	0.8887	0.0016	0.4855	0.047*	
C3	0.7952 (6)	−0.0832 (5)	0.3876 (5)	0.0516 (18)	
C4	0.6892 (6)	−0.0984 (5)	0.3507 (5)	0.0537 (19)	
H4	0.6725	−0.1482	0.3041	0.064*	
C5	0.6067 (6)	−0.0415 (5)	0.3812 (5)	0.0542 (18)	
H5	0.5354	−0.0530	0.3545	0.065*	
C6	0.6288 (5)	0.0312 (4)	0.4498 (5)	0.0382 (15)	
H6	0.5731	0.0697	0.4699	0.046*	
C7	0.8869 (7)	−0.1441 (6)	0.3487 (7)	0.089 (3)	
H7A	0.8624	−0.2075	0.3418	0.133*	
H7B	0.9488	−0.1409	0.4008	0.133*	
H7C	0.9068	−0.1220	0.2792	0.133*	
C8	0.5467 (4)	0.2559 (4)	0.8132 (4)	0.0289 (12)	
C9	0.5205 (5)	0.2946 (5)	0.7122 (5)	0.0475 (17)	
H9	0.5676	0.2878	0.6570	0.057*	
C10	0.4236 (6)	0.3440 (6)	0.6922 (6)	0.066 (2)	
H10	0.4070	0.3706	0.6239	0.080*	
C11	0.3525 (6)	0.3539 (5)	0.7720 (7)	0.061 (2)	
H11	0.2886	0.3878	0.7586	0.073*	
C12	0.3767 (5)	0.3133 (5)	0.8715 (6)	0.0499 (18)	
H12	0.3278	0.3180	0.9253	0.060*	
C13	0.4728 (5)	0.2655 (4)	0.8929 (5)	0.0394 (15)	
H13	0.4887	0.2392	0.9614	0.047*	
C14	0.8439 (5)	0.2784 (4)	0.8187 (4)	0.0299 (13)	
C15	0.8425 (5)	0.3467 (4)	0.7412 (5)	0.0439 (16)	
H15	0.7786	0.3574	0.6977	0.053*	
C16	0.9327 (6)	0.4001 (5)	0.7254 (6)	0.0566 (19)	
H16	0.9288	0.4471	0.6731	0.068*	
C17	1.0270 (6)	0.3841 (5)	0.7861 (6)	0.058 (2)	
H17	1.0883	0.4196	0.7750	0.070*	
C18	1.0322 (5)	0.3148 (5)	0.8646 (6)	0.0540 (19)	
H18	1.0969	0.3039	0.9066	0.065*	
C19	0.9419 (5)	0.2624 (4)	0.8804 (5)	0.0394 (15)	
H19	0.9460	0.2155	0.9329	0.047*	
C20	0.7300 (5)	0.0694 (4)	0.9337 (4)	0.0312 (13)	
C21	0.6583 (5)	0.0405 (4)	1.0097 (5)	0.0430 (16)	
H21	0.5969	0.0757	1.0214	0.052*	
C22	0.6796 (7)	−0.0412 (5)	1.0677 (5)	0.057 (2)	
H22	0.6319	−0.0607	1.1182	0.069*	
C23	0.7689 (8)	−0.0929 (5)	1.0516 (6)	0.066 (2)	
H23	0.7818	−0.1477	1.0908	0.079*	
C24	0.8394 (7)	−0.0651 (5)	0.9787 (7)	0.065 (2)	
H24	0.9013	−0.1003	0.9690	0.077*	
C25	0.8198 (5)	0.0154 (4)	0.9186 (5)	0.0472 (17)	
H25	0.8678	0.0332	0.8675	0.057*	

### Atomic displacement parameters (Å^2^)

**Table:**

	*U*^11^	*U*^22^	*U*^33^	*U*^12^	*U*^13^	*U*^23^
Sn1	0.0338 (2)	0.0285 (2)	0.0204 (2)	0.00111 (18)	0.00173 (15)	0.00267 (18)
Se1	0.0344 (3)	0.0338 (3)	0.0231 (3)	0.0015 (3)	0.0036 (2)	−0.0007 (3)
O1	0.053 (3)	0.039 (2)	0.018 (2)	0.009 (2)	0.0011 (18)	−0.0015 (18)
O2	0.053 (3)	0.038 (2)	0.016 (2)	−0.007 (2)	0.0051 (17)	−0.0003 (18)
C1	0.050 (4)	0.026 (3)	0.015 (3)	0.003 (3)	0.007 (2)	0.000 (2)
C2	0.045 (4)	0.041 (3)	0.032 (3)	0.010 (3)	0.004 (3)	0.002 (3)
C3	0.077 (5)	0.047 (4)	0.032 (4)	0.021 (4)	0.014 (4)	−0.004 (3)
C4	0.086 (6)	0.043 (4)	0.033 (4)	−0.009 (4)	0.005 (4)	−0.004 (3)
C5	0.059 (5)	0.059 (4)	0.045 (4)	−0.003 (4)	0.000 (3)	0.000 (4)
C6	0.038 (4)	0.035 (3)	0.042 (4)	−0.001 (3)	0.005 (3)	−0.002 (3)
C7	0.106 (7)	0.092 (7)	0.070 (6)	0.040 (6)	0.017 (5)	−0.030 (5)
C8	0.034 (3)	0.023 (3)	0.029 (3)	0.001 (3)	0.000 (2)	−0.001 (3)
C9	0.047 (4)	0.065 (4)	0.032 (4)	0.012 (3)	0.009 (3)	0.010 (3)
C10	0.058 (5)	0.091 (6)	0.050 (5)	0.022 (4)	0.003 (4)	0.030 (4)
C11	0.042 (4)	0.070 (5)	0.070 (5)	0.022 (4)	0.003 (4)	0.003 (4)
C12	0.036 (4)	0.066 (5)	0.050 (4)	−0.001 (3)	0.014 (3)	−0.015 (4)
C13	0.038 (4)	0.050 (4)	0.031 (3)	−0.002 (3)	0.008 (3)	0.001 (3)
C14	0.037 (3)	0.030 (3)	0.024 (3)	0.001 (2)	0.009 (2)	−0.004 (3)
C15	0.051 (4)	0.049 (4)	0.031 (4)	0.000 (3)	−0.005 (3)	0.008 (3)
C16	0.064 (5)	0.057 (4)	0.051 (5)	−0.011 (4)	0.017 (4)	0.012 (4)
C17	0.048 (4)	0.067 (5)	0.063 (5)	−0.018 (4)	0.018 (4)	0.005 (4)
C18	0.030 (4)	0.066 (5)	0.065 (5)	0.001 (3)	−0.003 (3)	−0.009 (4)
C19	0.038 (4)	0.039 (3)	0.041 (4)	0.006 (3)	0.002 (3)	0.003 (3)
C20	0.046 (4)	0.028 (3)	0.018 (3)	−0.003 (3)	−0.003 (2)	0.000 (2)
C21	0.056 (4)	0.039 (4)	0.034 (4)	−0.009 (3)	0.007 (3)	0.000 (3)
C22	0.089 (6)	0.053 (4)	0.030 (4)	−0.025 (4)	0.001 (4)	0.011 (3)
C23	0.103 (7)	0.040 (4)	0.051 (5)	−0.002 (4)	−0.015 (5)	0.018 (4)
C24	0.081 (6)	0.042 (4)	0.069 (5)	0.022 (4)	−0.008 (4)	−0.005 (4)
C25	0.056 (4)	0.040 (4)	0.046 (4)	0.010 (3)	0.002 (3)	0.003 (3)

### Geometric parameters (Å, °)

**Table:**

Sn1—C8	2.119 (5)	C10—H10	0.9300
Sn1—C20	2.132 (5)	C11—C12	1.367 (10)
Sn1—C14	2.136 (5)	C11—H11	0.9300
Sn1—O2	2.197 (4)	C12—C13	1.378 (9)
Sn1—O1^i^	2.273 (4)	C12—H12	0.9300
Se1—O1	1.674 (4)	C13—H13	0.9300
Se1—O2	1.698 (3)	C14—C15	1.367 (8)
Se1—C1	1.928 (5)	C14—C19	1.397 (8)
O1—Sn1^ii^	2.273 (4)	C15—C16	1.376 (9)
C1—C2	1.376 (7)	C15—H15	0.9300
C1—C6	1.391 (8)	C16—C17	1.354 (10)
C2—C3	1.384 (9)	C16—H16	0.9300
C2—H2	0.9300	C17—C18	1.383 (10)
C3—C4	1.368 (10)	C17—H17	0.9300
C3—C7	1.533 (9)	C18—C19	1.369 (9)
C4—C5	1.378 (9)	C18—H18	0.9300
C4—H4	0.9300	C19—H19	0.9300
C5—C6	1.356 (9)	C20—C25	1.376 (8)
C5—H5	0.9300	C20—C21	1.396 (8)
C6—H6	0.9300	C21—C22	1.386 (9)
C7—H7A	0.9600	C21—H21	0.9300
C7—H7B	0.9600	C22—C23	1.354 (10)
C7—H7C	0.9600	C22—H22	0.9300
C8—C9	1.376 (8)	C23—C24	1.356 (10)
C8—C13	1.393 (8)	C23—H23	0.9300
C9—C10	1.395 (9)	C24—C25	1.382 (9)
C9—H9	0.9300	C24—H24	0.9300
C10—C11	1.371 (10)	C25—H25	0.9300
			
C8—Sn1—C20	123.1 (2)	C9—C10—H10	119.6
C8—Sn1—C14	119.4 (2)	C12—C11—C10	119.2 (6)
C20—Sn1—C14	117.1 (2)	C12—C11—H11	120.4
C8—Sn1—O2	92.10 (18)	C10—C11—H11	120.4
C20—Sn1—O2	91.81 (17)	C11—C12—C13	120.6 (6)
C14—Sn1—O2	92.88 (17)	C11—C12—H12	119.7
C8—Sn1—O1^i^	88.08 (18)	C13—C12—H12	119.7
C20—Sn1—O1^i^	85.07 (17)	C12—C13—C8	120.9 (6)
C14—Sn1—O1^i^	90.18 (17)	C12—C13—H13	119.5
O2—Sn1—O1^i^	176.38 (13)	C8—C13—H13	119.5
O1—Se1—O2	102.58 (19)	C15—C14—C19	117.5 (5)
O1—Se1—C1	101.3 (2)	C15—C14—Sn1	122.8 (4)
O2—Se1—C1	100.0 (2)	C19—C14—Sn1	119.8 (4)
Se1—O1—Sn1^ii^	133.3 (2)	C14—C15—C16	122.0 (6)
Se1—O2—Sn1	128.6 (2)	C14—C15—H15	119.0
C2—C1—C6	119.6 (5)	C16—C15—H15	119.0
C2—C1—Se1	118.5 (5)	C17—C16—C15	119.8 (7)
C6—C1—Se1	121.8 (4)	C17—C16—H16	120.1
C1—C2—C3	121.1 (6)	C15—C16—H16	120.1
C1—C2—H2	119.4	C16—C17—C18	119.9 (6)
C3—C2—H2	119.4	C16—C17—H17	120.0
C4—C3—C2	117.9 (6)	C18—C17—H17	120.0
C4—C3—C7	120.9 (7)	C19—C18—C17	120.0 (6)
C2—C3—C7	121.1 (7)	C19—C18—H18	120.0
C3—C4—C5	121.5 (7)	C17—C18—H18	120.0
C3—C4—H4	119.3	C18—C19—C14	120.8 (6)
C5—C4—H4	119.3	C18—C19—H19	119.6
C6—C5—C4	120.4 (7)	C14—C19—H19	119.6
C6—C5—H5	119.8	C25—C20—C21	118.7 (6)
C4—C5—H5	119.8	C25—C20—Sn1	121.2 (4)
C5—C6—C1	119.4 (6)	C21—C20—Sn1	120.1 (4)
C5—C6—H6	120.3	C22—C21—C20	119.3 (6)
C1—C6—H6	120.3	C22—C21—H21	120.3
C3—C7—H7A	109.5	C20—C21—H21	120.3
C3—C7—H7B	109.5	C23—C22—C21	120.9 (7)
H7A—C7—H7B	109.5	C23—C22—H22	119.5
C3—C7—H7C	109.5	C21—C22—H22	119.5
H7A—C7—H7C	109.5	C22—C23—C24	120.2 (7)
H7B—C7—H7C	109.5	C22—C23—H23	119.9
C9—C8—C13	118.2 (5)	C24—C23—H23	119.9
C9—C8—Sn1	119.6 (4)	C23—C24—C25	120.3 (7)
C13—C8—Sn1	121.7 (4)	C23—C24—H24	119.9
C8—C9—C10	120.2 (6)	C25—C24—H24	119.9
C8—C9—H9	119.9	C20—C25—C24	120.6 (7)
C10—C9—H9	119.9	C20—C25—H25	119.7
C11—C10—C9	120.8 (7)	C24—C25—H25	119.7
C11—C10—H10	119.6		

Symmetry codes: (i) *x*, −*y*+1/2, *z*+1/2; (ii) *x*, −*y*+1/2, *z*−1/2.

### Hydrogen-bond geometry (Å, °)

**Table:**

*D*—H···*A*	*D*—H	H···*A*	*D*···*A*	*D*—H···*A*
C9—H9···O1	0.93	2.57	3.487 (7)	169.
